# Health Tract, No. 207

**Published:** 1864-06

**Authors:** 


HEALTH TRACT, No. 207.

### INHERITANCES.

On the last Sabbath of the last year, that good old minister McElroy, rose in his place and said: "I have been preaching to you forty years this day. Of all the elders who then held up my hands, not one survives; of all the male members, not one remains; of all the women, only six live. But although the fathers and mothers have passed away, the more numerous sons and daughters have taken their places, and their children's children are convincing evidences that He is a covenant-keeping God whom we are serving this day, in that the grand-children, having had a good example set before them of holy living, of Sabbath observance, of habitual attendance on the services of the sanctuary, and a profound reverence, with an unquestioning and blessed faith in the word of God, have become religious by inheritance, as it were."

Sons have often inherited the wealth of their fathers, even to the third and fourth generation. The same principle holds good as to our physical nature, that a life of temperance and industry and moderate ambitions secures to children, even for several generations, a robustness of constitution, a vitality, a physical power, which may well be the envy of a multitude of the sick and diseased and effeminate in every grade of society. Children who see daily in their parents the practice of all that is gentle and lovable and courteous and kind, will seldom fail, without the necessity of direct teachings on these subjects, to acquire the same traits of character; and the example lives and has its influence and power for good long after the parents have passed away. If parents want their children to grow up and inherit their own robust health, strength, and length of life, it must come, not so much by birth and blood, not so much by precept and command and reason, but by the daily exhibition of a calm, quiet, busy, temperate life on the part of their parents, carried out daily, habitually, and persistently by living examples. Conduct is the great, efficient teacher, not precept, not theory, not idle profession.

